# Fibroblast growth factor receptor 3 gene (FGFR3) mutations in high-grade muscle-invasive urothelial bladder cancer in a Brazilian population: evaluation and prevalence

**DOI:** 10.31744/einstein_journal/2022AO6450

**Published:** 2022-03-22

**Authors:** Camila Ribeiro de Arruda Monteiro, Fernando Korkes, Deborah Krutman-Zveibil, Sidney Glina

**Affiliations:** 1 Centro Universitário FMABC Santo André SP Brazil Centro Universitário FMABC, Santo André, SP, Brazil.

**Keywords:** Urinary bladder neoplasms, Receptor, fibroblast growth factor, type 3, Mutation, Polymerase chain reaction, DNA, Sequence analysis, DNA, Carcinoma, transitional cell

## Abstract

**Objective:**

To understand the feasibility of FGFR3 tests in the Brazilian public health context, and to sample the mutational burden of this receptor in high-grade muscle invasive bladder cancer.

**Methods:**

A total of 31 patients with high-grade muscle-invasive bladder cancer were included in the present study. Either transurethral resection of bladder tumor or radical cystectomy specimens were analyzed. Formalin-fixed paraffin-embedded tissue blocks were sectioned, hematoxylin and eosin stained, and histologic sections were reviewed. Total RNA was extracted using the RNeasy DSP formalin-fixed paraffin-embedded kit. Qualitative results were displayed in Rotor-Gene AssayManager software.

**Results:**

Six patients were excluded. From the samples analyzed, four (16.7%) were considered inadequate and could not have their RNA extracted. Two patients presented FGFR3 mutations, accounting for 9.5% of material available for adequate analysis. The two mutations detected included a Y373C mutation in a male patient and a S249C mutation in a female patient.

**Conclusion:**

FGFR3 mutations could be analyzed in 84% of our cohort and occurred in 9.5% of patients with high-grade muscle invasive bladder cancer in this Brazilian population. FGFR3 gene mutations are targets for therapeutic drugs in muscle-invasive bladder cancer. For this reason, know the frequency of these mutations can have a significant impact on public health policies and costs provisioning.

## INTRODUCTION

Bladder cancer has a high mutational burden, and fibroblast growth factor receptor 3 gene (FGFR3) is one of the most frequently mutated genes in bladder cancer. It is reported that more than 65% of non-muscle invasive bladder cancer (NMIBC) carry FGFR3 mutations.^(1)^ A large study evaluated 10,032 bladder cancer and identified 56 different FGFR3 mutations.^(2)^

The fibroblast growth factor (FGF) family of transmembrane tyrosine kinase receptors mediates proliferation in response to FGF stimulation and has been implicated in the pathogenesis of urothelial cancer. Fibroblast growth factor receptor 1 gene (FGFR1) and FGFR3 are either mutated or overexpressed in the majority of NMIBC.^(3)^ These mutations are less frequently observed in muscle-invasive bladder cancer (MIBC) and less common in high-grade tumors than in low-grade tumors.^(4)^

It can be vital to understand the FGFR3 mutational burden of MIBC, since there are therapeutic agents with clinical benefits for this specific situation.^(5)^ Additionally, patients with FGFR3 alterations tend to have a low likelihood of response to chemotherapy and PD-1 and PD-L1 inhibitors.^(5-7)^ FGFR3 mutations are currently targets for therapeutic agents in bladder cancer. Erdafinib has already been approved by the Food and Drug Administration (FDA) for metastatic bladder cancer after first-line therapy. However, this agent is only approved if an FGFR3 mutation is detected.^(5)^

The access to comprehensive genomic tests in Brazil is still limited, and the prevalence of FGFR3 mutations among Brazilian MIBC patients is not well known. Brazil is a country with a sizeable territorial extension, and some degree of genetic background heterogeneity may exist in the population.^(8)^Precision medicine and targeted therapies are already a reality in the pipeline of bladder cancer treatment.^(9)^ In such a context, knowledge of the molecular profile of MIBC in Brazil is fundamental to define better public health strategies, for it can have major impacts.^(10)^

## OBJECTIVE

To understand the feasibility of FGFR3 tests in the Brazilian public health context, and to sample the mutational burden of this receptor in high-grade muscle invasive bladder cancer.

## METHODS

From May 2020 to November 2020 a total of 31 patients with high-grade MIBC were included in the present study. Either transurethral resection of bladder tumor (TURBT) or radical cystectomy specimens were available for analysis.

Formalin-fixed paraffin-embedded (FFPE) tissue blocks with tumor samples were previously identified. All hematoxylin and eosin stained histologic sections from specimens were reviewed. Index tumor (highlighted on slides) was defined as focusing on the worst pattern and area with muscle-invasion. Urothelial carcinomas were staged according to the Union for International Cancer Control (UICC) criteria, and graded according to World Health Organization (WHO) criteria.

The Institutional Medical Ethics Committee of the *Faculdade de Medicina do ABC* (FMABC) approved the present study, CAAE: 31079120.6.0000.0082, protocol # 4.039.916, and all patients signed the Informed Consent Form.

### Genomic RNA

As per the manufacturer protocols (Qiagen, Hilden, Germany), total RNA was extracted from FFPE urothelial tumor samples using the RNeasy DSP FFPE kit. Purified RNA was then reversely transcribed using reverse transcriptase to generate cDNA for real-time PCR analysis. Qualitative results were displayed in Rotor-Gene AssayManager software, informing the system operator if one or more of the four-point mutations and two fusions in the FGFR3 gene detected by the kit were present in each sample. The therascreen FGFR RGQ RT-PCR kit is a qualitative *in vitro* diagnostic test for the detection of two point mutations in exon 7 – p.R248C (c.742C>T) and p.S249C (c.746C>G) –, two-point mutations in exon 10 – p.G370C (c.1108G>T) and p.Y373C (c.1118A>G) –, and two fusions – FGFR3*-TACC3v1* and FGFR3*-TACC3v3* – in the FGFR3 gene.^(11)^ Fibroblast growth factor receptor mutations tested included R248C, G370C, S249C, Y373C, TAC3V3, BAIAP2L1, CASP7, TACC3V1, and BICC1.

### Statistical analysis

Data were analyzed using STATA 14.0 (StataCorp LP, College Station, Texas, United States). Frequency tables were chosen for descriptive analyses. Chi-squared and Fisher’s exact test were chosen to assess the frequency of responses among groups. For continuous variables, we used the Mann-Whitney test, and p<0.05 was considered significant.

## RESULTS

### Patients and tumors

A total of 31 patients were included in the present study. All patients had a histologic diagnosis of high-grade muscle-invasive urothelial carcinoma of the urinary bladder. Patients’ demographics are presented in table 1. Of the total, two patients refused to continue on the study, and four could not be contacted to give consent.

**Table 1:** Patients’ demographics

Demographics	
Age, years	65.0±8.2
BMI, kg/m^2^	27.0±4.4
Ethnicity	
White	17 (68)
Black	1 (4)
Others	7 (28)
Sex	
Male	14 (56)
Female	11 (44)
Smoking status	
Smoker	9 (36)
Former smoker	8 (32)
2Never smoked	6 (24)
Unknown	2 (8)
T staging	
2	18 (72)
3	4 (16)
4	3 (12)
N staging	
Negative	19 (76)
Positive	6 (24)
M staging	
Negative	21 (84)
Positive	4 (16)
Serum hemoglobin, g/dL	13.1±2.2
Creatinine, mg/dL	1.1±0.7
Creatinine clearence, mL/min/1.73m^2^	56.0±30.2

Results expressed as media ± standard deviation or n (%).

BMI: body mass index.

### FGFR3 mRNA analysis and mutations

**Figure 1:**
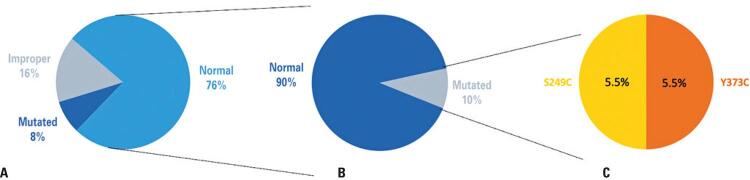
Tests results and FGFR3 mutations

From the samples analyzed, four (16.7%) were considered inadequate samples and could not have their RNA extracted, remaining 21 patients for further analysis. Two of the patients presents FGFR3 mutations, representing 9.5% of material available for adequate analysis. The two mutations detected included a Y373C mutation in a male patient, and a S249C mutation in a female patient. These results are represented in figure 1.

## DISCUSSION

It is known that FGFR3 mutations are common in bladder cancer. However, they tend to occur more frequently in non-muscle invasive and low-grade tumors. It is less common in muscle-invasive and high-grade tumors. FGFR3 mutations are less common in these patients, but this population arouses great interest. They have a higher risk of developing metastatic disease. In recent years, FGFR3 inhibitors, such as erdafitinib, have been approved by the FDA for patients with metastatic urothelial carcinoma after first-line treatment. Therefore, our study evaluated the expression of FGFR3 mutations in patients with high-grade MIBC, in a Brazilian sample.

We had some interesting findings. First, the frequency of FGFR3 mutation in our cohort was relatively low, with 9.5% in the tumors analyzed. Although our sample size was small, we found results very similar to those from other studies.^(12)^ Gust et al., also reported FGFR3 mutations in 11% of patients, in a similar cohort from Europe, with Greek and Spanish patients.^(13)^ Another study found 6% of mutations.^(14)^These numbers are much fewer than those found in non-invasive and low-grade bladder cancer.^(4)^

Previous studies identified the S249C mutation (TCC→TGC), which accounts for 62% of all recurrent FGFR3 mutations, and Y373C as the second most frequent, accounting for approximately 29% of all mutations.^(15-18)^ The Y373C and the S249C mutations were found in the present study. They are Apolipoprotein B mRNA Editing Catalytic Polypeptide-like (APOBEC)-mediated mutations, what might explain their overrepresentation in bladder cancer as compared to other FGFR3 alterations.^(2)^ Our sample size was small and we could identify only these two cases; however, this finding is in accordance with those of larger cohorts. Genetic mutations can vary among different populations.^(8)^ For this reason, it is crucial to analyze a Brazilian sample.

Our study has several limitations. First, our cohort of patients is small. Additionally, mutation analysis of FGFR3 was not performed in FFPE tissue. Tumor heterogeneity and processing limitations were responsible for some limitations of this method. Nonetheless, it was possible to validate externally with other cohorts of patients, demonstrating similar results. Thus, our observation that 9.5% of high-grade MIBC sampled from the primary tumor had FGFR3 mutations is reasonable based on the published literature.

Additionally, four of our cases could not be examined due to technical limitations. In 16.7% of cases, the sample was inadequate for RNA extraction. Our study was performed in FFPE tissue. It is known that this material might have some limitations.^(19)^ Some variables, such as cold ischemia time, quality of formalin (10%, buffered), paraffin quality, and storage conditions are critical.^(20)^ These aspects of the analysis are cornerstones for precision medicine. Biomolecular markers rely on good-quality material. Our study demonstrates that we have much to improve in this field. A third of our samples were rendered improper for further RNA extraction and could not be analyzed. When a TURBT is performed, details such as the cautery configuration or the amount of time to store the tissue in the formalin, can significantly impact this specific outcome.

## CONCLUSION

FGFR3 gene mutations could be analyzed in 84% of our cohort, and they occurred in 9.5% of patients with high-grade muscle invasive bladder cancer in this Brazilian population. FGFR3 gene mutations are targets for therapeutic drugs in muscle invasive bladder cancer. For this reason, know the frequency of these mutations can have a significant impact on public health policies and costs provisioning.
